# TYMS Enhances Colorectal Cell Antioxidant Capacity Via the KEAP1-NRF2 Pathway to Resist Ferroptosis

**DOI:** 10.7150/jca.102931

**Published:** 2025-01-01

**Authors:** Jingtian Chen, Wei Wu, Lingxiao Wang, Jingjing Zhang, Xin Che, Liqin Zhai, Zhenxiang Zhao, Yaoping Li

**Affiliations:** 1 The Colorectal and Anal Surgery Department of Shanxi Provincial People's Hospital, Shanxi Medical University, Taiyuan, China.; 2 Translational Medicine Research Center, Shanxi Medical University, Taiyuan, China.

**Keywords:** Thymidylate synthase, Nuclear factor erythroid 2-related factor 2, Kelch-like ECH-associated protein 1, ferroptosis, ROS, Colorectal cancer

## Abstract

**Purpose:** Thymidylate synthase (TYMS) is a key regulatory enzyme in DNA synthesis. We identified the biological effect and molecular mechanisms of TYMS in colorectal cancer (CRC).

**Methods:** We employed western blot and immunohistochemistry for the assessment of TYMS expression in CRC samples. MTT and colony assay were carried out to illuminate the effect of TYMS on the proliferation of CRC cells. Xenograft models were constructed to evaluate the consequences of TYMS overexpression on CRC *in vivo*. Metabolomics was utilized to analyze the alterations in cellular molecular metabolites subsequent to TYMS overexpression. The impact of TYMS on NRF2 localization and KEAP1 expression was explored by means of western blot. The expression levels of GSH, ROS, MDA, and *PTGS2* mRNA were measured to assess ferroptosis.

**Results:** TYMS expression in CRC tumor tissues was upregulated compared to adjacent non-cancerous tissues. Cells overexpressing TYMS displayed enhanced proliferative capabilities. Metabolomic analysis revealed that overexpression of TYMS was associated with elevated levels of GSH within cells and a decrease in the lipid peroxidation product, 4-hydroxyhexenal. ROS detection assays further demonstrated a significant enhancement in cellular antioxidant capacity due to TYMS overexpression. Overexpression of TYMS downregulated KEAP1 expression and promoted NRF2 translocation into the nucleus. Consequently, transcription of downstream antioxidant genes was upregulated, enhancing cellular antioxidant capacity, reducing ROS levels, diminishing lipid peroxidation products, and heightening resistance to ferroptosis induced by erastin. Additionally, our study indicated that the TYMS inhibitor 5-fluorouracil (5-FU) exhibited favorable drug synergism with erastin.

**Conclusion:** TYMS was overexpressed in CRC, which was correlated with poor prognosis of CRC patients. TYMS enhanced the antioxidant capacity of CRC cells via the KEAP1-NRF2 pathway, thereby increasing resistance to erastin-induced ferroptosis.

## Introduction

In the 2022 global cancer statistics, colorectal cancer (CRC) stood as the third most prevalent cancer and the second leading cause of death^1^. With a lack of early diagnostic tumor markers, many patients are diagnosed at advanced stages, thus constraining treatment options.

Thymidylate synthase (TYMS) is a key regulatory enzyme in DNA synthesis, utilizing 5,10-methylenetetrahydrofolate (5,10-CH2-THF) as a methyl donor to methylate deoxyuridine monophosphate (dUMP) to deoxythymidine monophosphate (dTMP). dTMP functions as the primary source of thymidine within cellular environments, subsequently undergoing phosphorylation to form deoxythymidine triphosphate (dTTP), which constitutes one among four indispensable building blocks required for DNA replication^2^. Targeting TYMS has emerged as one of the most successful therapeutic strategies^3^. Among these approaches, 5-fluorouracil (5-FU) stands out as a foundational drug, forming the core of the mFOLFOX6 regimen when combined with oxaliplatin, which is the preferred chemotherapy protocol in most national CRC treatment guidelines^4^. However, genetic polymorphisms of TYMS and tumor heterogeneity contribute to 5-FU resistance in certain patients, diminishing the efficacy of chemotherapy. Investigating mechanisms of drug resistance and exploring multi-targeted combination therapies are critical areas of current research endeavors^5^, ^6^.

Ferroptosis is a form of cell death driven by iron-dependent lipid peroxidation^7^. Current research has established a connection between ferroptosis and various human diseases, presenting a promising avenue for cancer treatment^8^, ^9^. Unlike apoptosis, necroptosis, and pyroptosis, ferroptosis is characterized by metabolic imbalance and is the result of the collapse of the cell's antioxidant system^10^. Metabolic reprogramming stands as one of the key features of tumor cells^11^. In order to meet their rapid proliferation requirements, tumor cells undergo energy metabolism accompanied by the generation of abundant reactive oxygen species (ROS). Excessive production of ROS can trigger lipid peroxidation on cellular membranes, impacting phospholipids, glycolipids, and unsaturated fatty acids, resulting in disturbances in lipid bilayer structure and ultimately accelerating ferroptosis in cells^12^. The KEAP1-NRF2 system serves as a crucial regulator of the antioxidant system^13^. KEAP1 binds to NRF2 in the cytoplasm, leading to NRF2 ubiquitination and degradation^14^. Under certain specific conditions, NRF2 dissociates from KEAP1 and translocates into the nucleus, thereby promoting the transcription of downstream antioxidant genes, reducing lipid peroxidation occurrence, and conferring resistance against cell ferroptosis^15^-^17^. Our investigation revealed that upregulating TYMS expression in CRC cells led to diminished KEAP1 expression, facilitating NRF2 translocation into nucleus and increasing cellular resistance against erastin-induced ferroptosis.

## Materials and Methods

### Clinical samples

The CRC tissue microarray (TMA) was generously provided by the Pathology Department of Shanxi Provincial People's Hospital. The study encompassed 173 cases, comprising CRC tissues with clinical pathological parameters and their paired adjacent non-cancerous tissues, with one case missing information. Among the participants, there were 97 males and 76 females, ranging in age from 32 to 85 years, with a median age of 62 years. The follow-up period extended over 10 years. All clinical samples used in this study were obtained with informed consent from the patients.

### Cell culture

CRC cell lines, namely SW480, SW620, HT29, HCT8, HCT116, and LOVO, were obtained from Procell (Wuhan, China). The overexpression and knockdown of TYMS were designed by Shanghai Jikai Gene Medicine Technology Co., Ltd. TYMS-specific sequences were cloned into the Ubi-MCS-flag-CMV-IRES-Puro lentiviral expression vector, denoted as TYMS, with the empty vector designated as vector. The knockdown sequence 5-CAACCCTGACGACAGAAGA-3, designated as shTYMS, and the negative control virus sequence 5-TTCTCCGAACGTGTCACGT-3, named shcon. The experimental drugs included MG132, ML385, erastin, Ferrostatin-1 and Z-VAD-FMK were all sourced from AbMole (USA).

### Animal experiments

Female BALB/c-Nude mice, aged four weeks (Gempharmatech, Jiangsu, China), were utilized for xenograft experiments. A mixture of 5×10^^6^ cells combined with PBS and Matrigel (Corning, USA) was subcutaneously injected into the right groin region of nude mice. Tumor volumes were measured using calipers with the formula: length (mm)×width (mm)²/2. All animal experiments were conducted in compliance with the National Research Council Guidelines for the Care and Use of Laboratory Animals and had received approval from the Ethics Committee of Shanxi Medical University (Approval Number: 2022SJL62).

### Immunohistochemical staining assay

Immunohistochemical staining was conducted on both the tissue microarray (TMA) and consecutive 4 μm paraffin-embedded sections for each xenograft tumor to evaluate the expression levels of specific proteins. All images were captured using the KF-PRO-005-EX microscope (Kfbio, China). Protein expression levels were analyzed using K-Viewer and ImageJ software.

### Cell proliferation assay

Methylthiazolyldiphenyl-tetrazolium bromide (MTT) was used to perform the cell viability assay. Cell suspensions were seeded into each well of a 96-well plate. After seeding, the plates were retrieved from the incubator at 24 h, 48 h, 72 h, 96 h, and 120 h. Subsequently, 20 μl of MTT solution (Solarbio, Beijing) was added to each well. Following a 4-hour incubation period, 100 μl of DMSO (Sigma, USA) was added. The absorbance at 570 nm of each well was measured using a Multimode Reader (Varioskan Flash, Thermo Electron Co., USA).

Colony-formation assay was used to perform the cell proliferation. 1000 cells were seeded in 2ml of serum-free culture medium per well in a six-well plate. Cultures were terminated when the number of single-cell colonies visible under the microscope exceeded 50. Subsequently, cells were stained with 0.25% crystal violet.

### RNA extraction and RT-qPCR

RNA extraction was conducted utilizing the RNAiso Kit (Takara, Japan). The isolated RNA was then reverse transcribed into cDNA using a reverse transcription kit (Takara, Japan). Real-time PCR (RT-qPCR) was performed using the SYBR Green method (Takara, Japan) on the StepOne Plus Real-time PCR system (Applied Biosystems). Gene expression levels were determined using the -2^ΔΔCT^ method. Primer sequences employed are detailed in Supplementary Table S1.

### Western blot

Cells were lysed in RIPA buffer (Solarbio, China) supplemented with protease inhibitor PMSF (Seven, China). Nuclear and cytoplasmic proteins were extracted using the Nuclear and Cytoplasmic Extraction Kit (Keygentec, China). 40 μg of total protein was electrophoresed on gels and subsequently transferred onto PVDF membranes. Membranes were then incubated overnight at 4°C with primary antibodies, followed by a 2-hour incubation with secondary antibodies at room temperature. Primary antibodies utilized, including TYMS, KEAP1, NRF2, β-actin, and PTGS2, were all sourced from Proteintech (Wuhan, China).

### Detection of ROS Levels

Cells were seeded in a 96-well opaque culture plate at a density of 2000 cells per well. The following day, 100 µL of DCFH-DA diluted solution (Beytime, China) was added to each well. The plate was then incubated at 37°C in cell culture incubator for 20 minutes. Fluorescence intensity was measured for each well using a Multimode Reader with excitation at 488 nm and emission at 525 nm.

### Detection of reduced glutathione (GSH) and oxidized glutathione (GSSG)

GSH and GSSG detection were conducted using a kit from Beytime (China) according to the manufacturer's protocol. GSSG levels were measured, and GSH levels were calculated using the formula: GSH = Total Glutathione - GSSG×2.

### Lipid peroxidation levels

Lipid peroxidation was assessed using the Malondialdehyde (MDA) content detection kit (Sangon, China) according to the manufacturer's protocol. Absorbance values at 600 nm and 532 nm were measured and utilized for calculation.

### Statistical analysis

Statistical analysis was conducted using SPSS 25.0 (Statistical Product and Service Solutions, USA) and Prism 8.0 (GraphPad Software, USA). Graphs and charts were generated using GraphPad Prism 8.0. The correlation between TYMS levels and clinical pathological characteristics was assessed using the chi-square test, while Kaplan-Meier analysis was employed to construct survival curves. Inter-group differences were evaluated using Student's t-test and analysis of variance (ANOVA), with significance set at *p* < 0.05.

## Results

### Elevated TYMS expression was correlated with a poor prognosis in CRC patients

**Figure 1:**
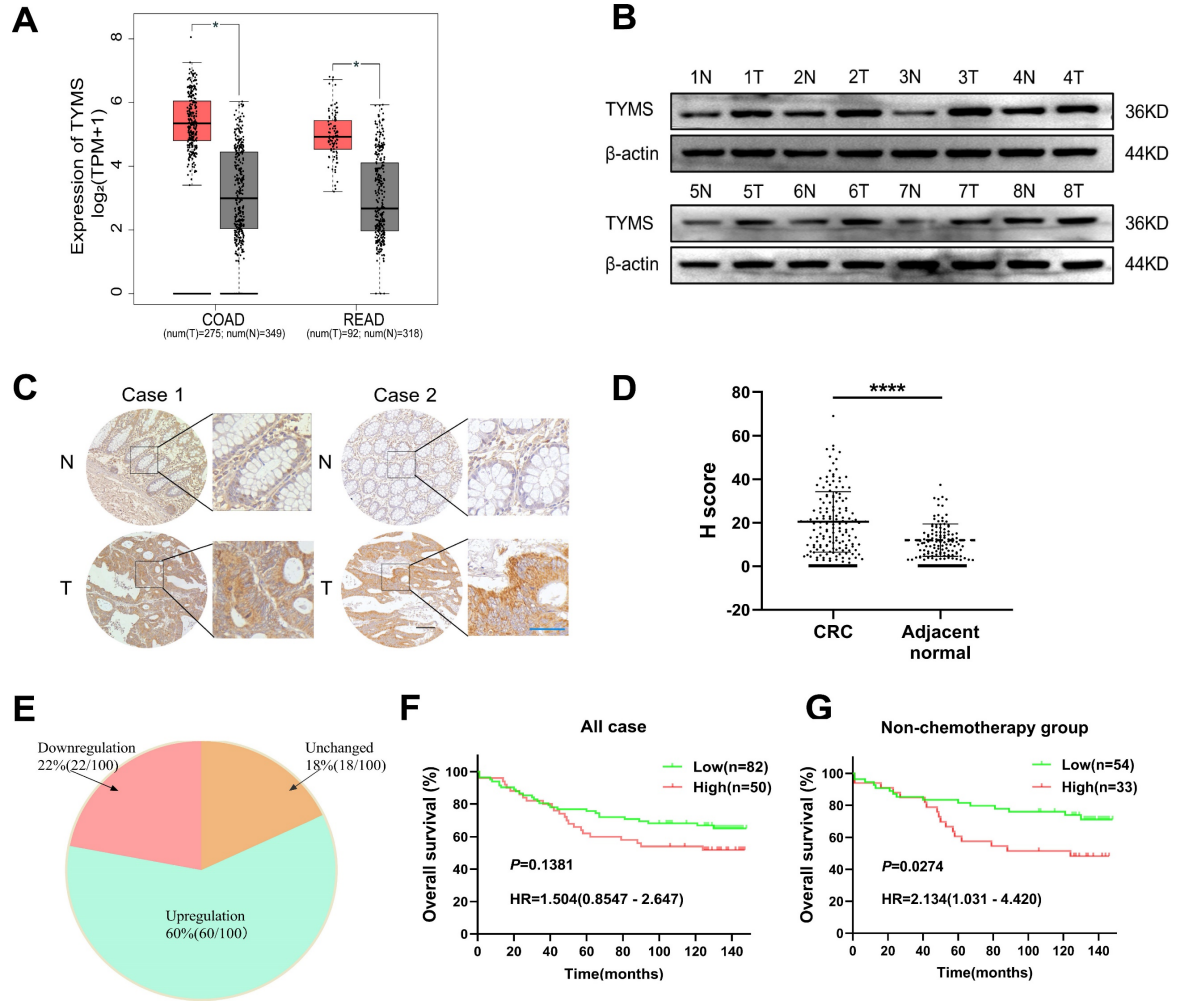
TYMS expression in CRC tissues and correlations to survival. (A) Expression of TYMS in CRC patients analyzed with the GEPIA online tool. (B) Detection of TYMS expression by western blot in CRC tissues and adjacent tissues from eight CRC patients. (C, D) Representative IHC images and the scoring of IHC showing the level of TYMS in CRC tissues and adjacent tissues. Black color scale bars: 100 μm, blue color scale bars: 50 μm. (E) Pie chart showing the proportion of upregulation, unchanged and downregulation in TYMS for comparison between CRC tissues and corresponding noncancerous tissues. *n*= 100 pair of samples. (F, G) Kaplan-Meier analysis of overall survival in all CRC patients and patients not receiving chemotherapy with differential TYMS expression. **** *p*< 0.0001.

Data retrieved from the GEPIA database revealed a significant upregulation of TYMS mRNA expression in colorectal and rectal cancer tissues compared to normal tissues (Figure 1A). After grinding CRC tissues and their paired adjacent tissues for Western blot analysis, the results demonstrated elevated expression of TYMS in CRC tissues compared to non-cancerous tissues (Figure 1B). Furthermore, we performed immunohistochemical staining on 173 clinically well-documented CRC tumor tissues and their corresponding adjacent tissues. Tissues lacking effective tumor structures or those with adjacent tissues lacking glandular structures were excluded from the statistical analysis. The immunohistochemical staining demonstrated TYMS expression in tumor tissues was markedly higher than adjacent tissues (Figure 1C, D), aligning with the conclusions drawn from the database analysis. Moreover, among 100 patients where TYMS protein expression was detectable in both the cancerous and adjacent tissues of the same patient, the upregulation rate of TYMS protein expression in CRC tumor tissues was 60% (60/100) (Figure 1E). Among the 132 patients with observable tumor tissues, the optimal cutoff value for immunohistochemical scoring was determined using X-tile (USA). Although patients with high TYMS expression tended to have a relatively poorer prognosis, the difference did not reach statistical significance (Figure 1F). Considering TYMS as the target of 5-FU, excluding patients who underwent chemotherapy, individuals with high TYMS expression exhibited a relatively poorer prognosis compared to those with low expression (Figure 1G). The expression levels of TYMS did not impact overall survival in patients with stage I/II or stage III/IV (Supplementary Figure 1). Additionally, high TYMS expression correlated with tumor size and N staging (Table 1).

### TYMS facilitated the proliferation of CRC cells

**Figure 2:**
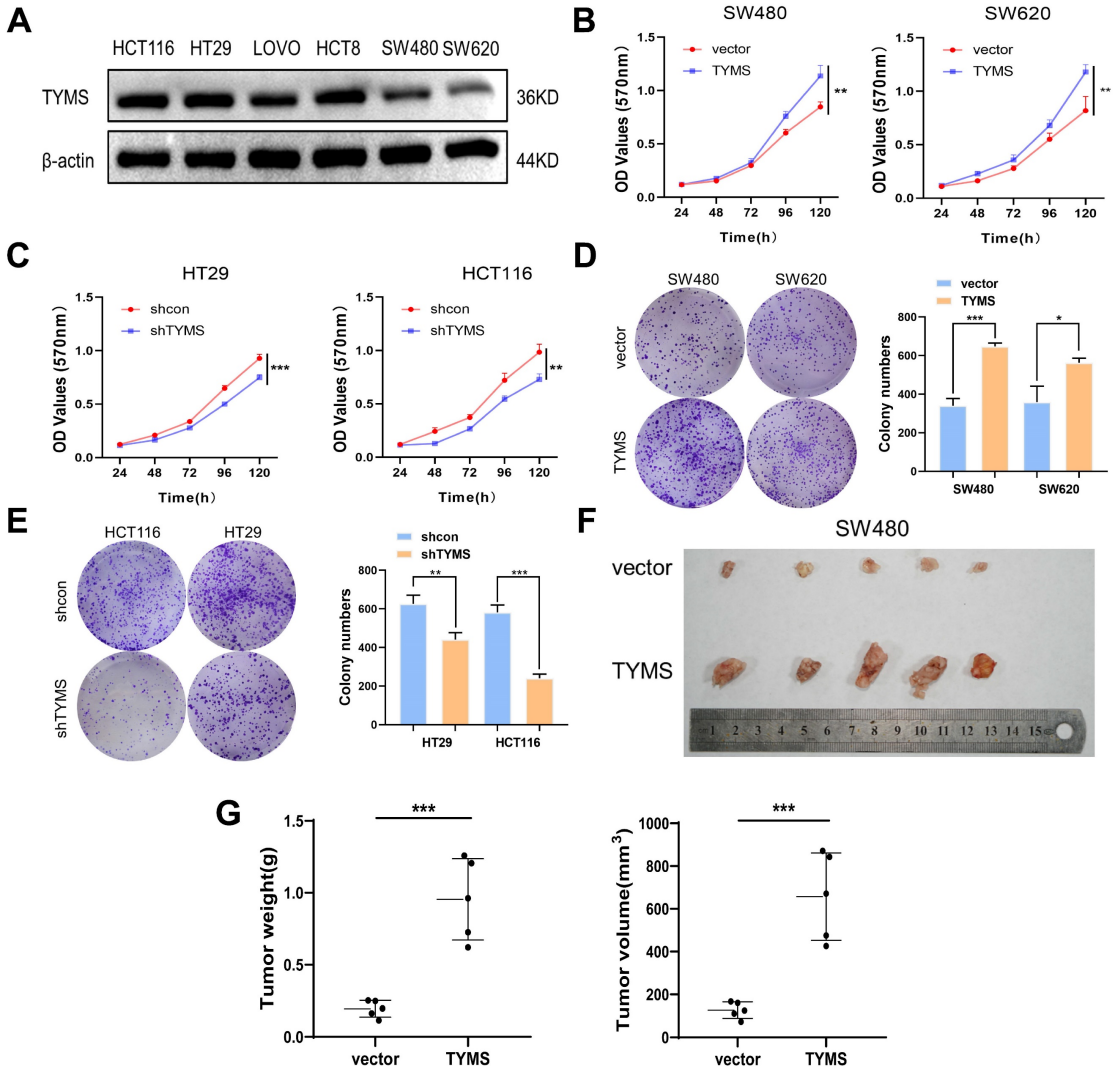
TYMS induced tumorigenesis. (A)Western blotting detected the expression of endogenous TYMS in different CRC cell lines. (B, C) Cell proliferation assay was performed in TYMS overexpressed cells (SW480 and SW620, B), knocked down cells (HT29 and HCT116, C), and correspondence control cells, respectively. (D, E) Colony formation assay was performed in TYMS overexpressed cells (D), TYMS knocked down cells (E), and correspondence control cells (vector or shcon). (F) Ectopic expressed TYMS in SW480 cells promoted tumor growth in xenograft mice model. (G) The volume and weight of tumors derived from TYMS ectopic expressed cells. *p < 0.05, **p < 0.01, ***p < 0.001.

To better elucidate the biological functions of TYMS in CRC, we examined the endogenous expression of TYMS in multiple CRC cell lines (Figure 2A). We chose HT29 and HCT116, characterized by higher endogenous expression, to establish stable TYMS knockdown cell lines. Stable TYMS overexpression cell lines were established in SW480 and SW620. Based on MTT assays and colony formation experiments, we observed that TYMS overexpression promoted both cell proliferation and colony formation (Figure 2B, D). In contrast, TYMS knockdown significantly inhibited both cell proliferation and colony formation (Figure 2C, E). Subsequently, we assessed the influence of TYMS on tumor formation ability through a xenograft mouse model. Mice injected with SW480 cells overexpressing TYMS exhibited accelerated malignant tumor formation compared to control groups (Figure 2F). The size and weight of the tumors in mice visually reflected the proliferative effect of TYMS (Figure 2G).

### TYMS enhanced the antioxidant capacity of CRC cells

**Figure 3:**
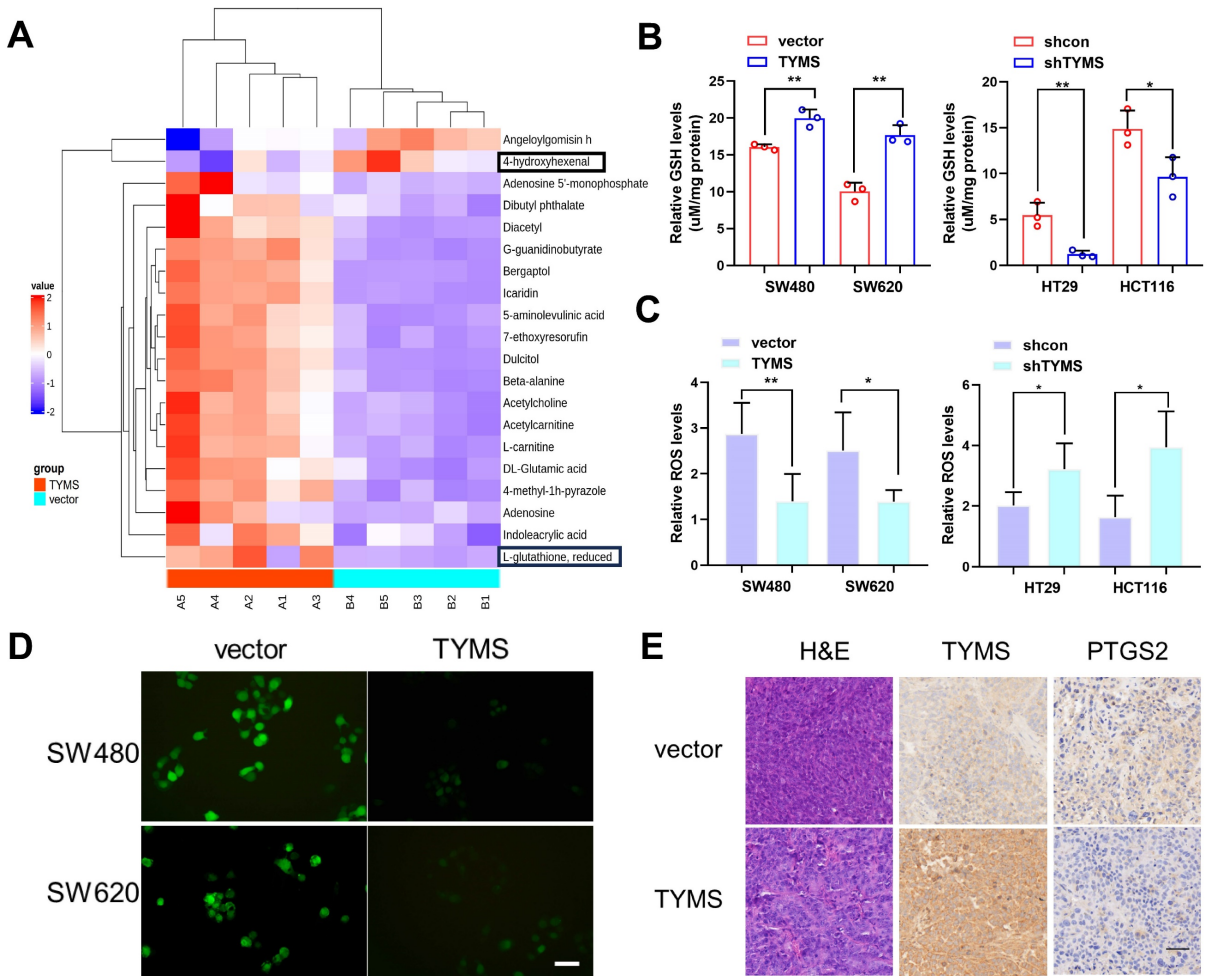
TYMS enhanced the antioxidant capacity. (A) Comparison of differential metabolites between TYMS overexpress (TYMS) and control cells (vector) in SW480 cell by untargeted Metabolomics. GSH levels(B) and ROS levels(C) in TYMS overexpressed cells (SW480 and SW620), knocked down cells (HT29 and HCT116), and correspondence control cells. (D) DCFH-DA fluorescent probe detected the fluorescence intensity of cells in TYMS overexpressed cells and correspondence control cells. Scale bars: 50 μm. (E) HE and IHC assay for TYMS and PTGS2 were performed in isolated tumor tissues. Scale bars: 100 μm. *p < 0.05, **p < 0.01.

To explore the mechanism through which TYMS affected the proliferation of CRC cells, we conducted metabolomic profiling on SW480 cells. The results showed that overexpression of TYMS in SW480 cells resulted in an elevation of GSH levels and a reduction in the content of the lipid peroxidation product, 4-hydroxyhexenal (Figure 3A). GSH is a critical endogenous antioxidant that functions to eliminate free radicals. Electrophilic aldehydes, including acrolein, 4-hydroxy-2-nonenal, and 4-hydroxyhexenal, are primary end products resulting from the oxidation of polyunsaturated fatty acids^18^. We postulated that TYMS enhanced the antioxidant capacity of CRC cells and resisted to ferroptosis, thereby promoting cell proliferation. GSH detection indicated that overexpression of TYMS led to an increase in GSH levels in SW480 and SW620 cells, while causing a decrease in GSH levels in HT29 and HCT116 cells (Figure 3B). The DCFH-DA assay kit, commonly employed for ROS detection^19^, was utilized in our study. Our findings indicated that overexpression of TYMS led to a significant decrease in ROS levels in SW480 and SW620 cells (Figure 3C, D). Conversely, TYMS knockdown resulted in substantial increase in the levels of ROS observed within HT29 and HCT116 cells (Figure 3C). This suggested that overexpression of the TYMS gene reduced ROS accumulation, thereby inhibiting oxidative stress in CRC cells. PTGS2 is a marker for evaluating ferroptosis *in vivo*. Histopathological examination (HE) along with immunohistochemistry assays were conducted on isolated tumor tissues focusing on TYMS and PTGS2 expression (Figure 3E). The PTGS2 level decreased with TYMS overexpression.

### TYMS promoted the nuclear translocation of NRF2, thereby enhancing the antioxidant capacity of CRC cells

**Figure 4:**
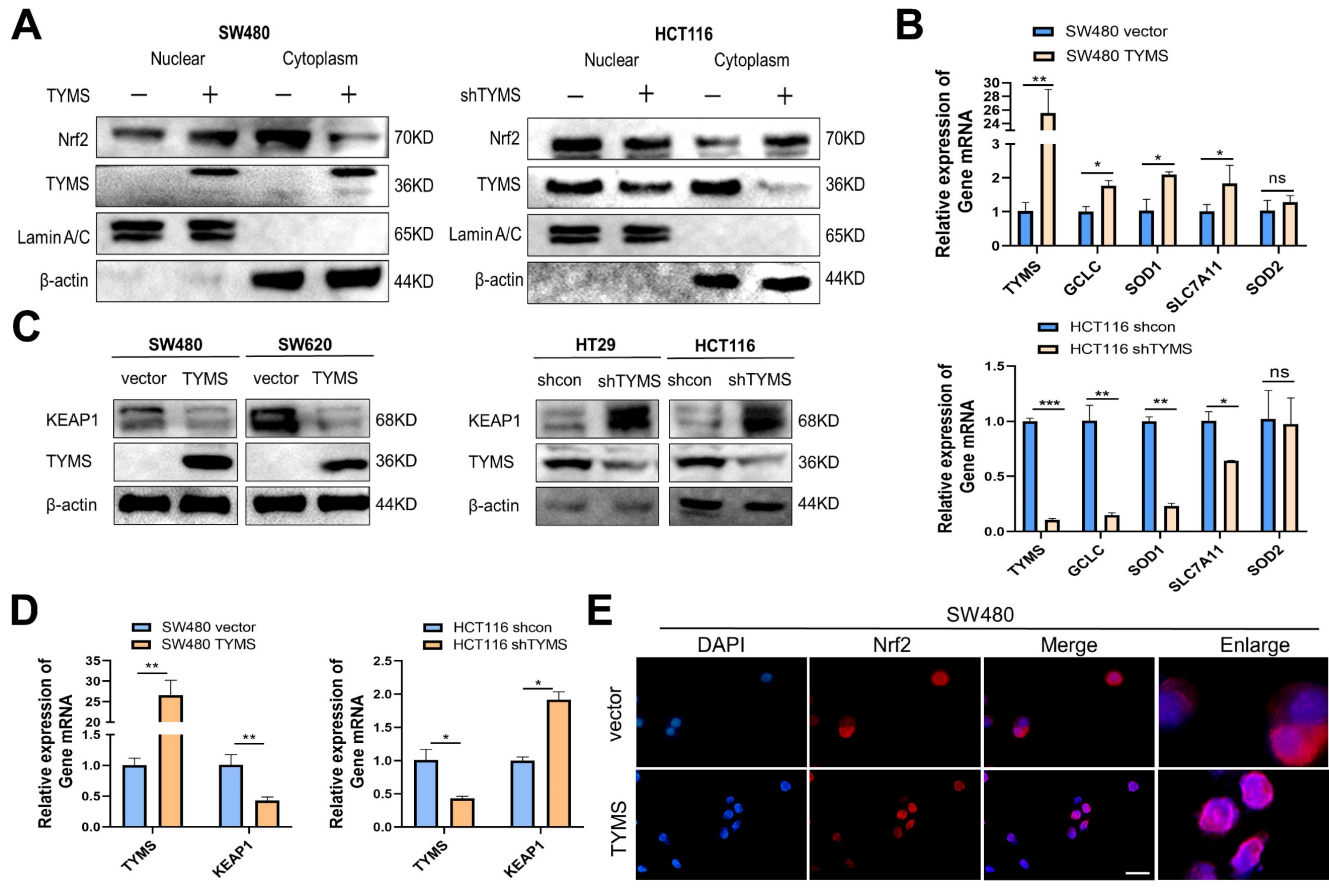
TYMS promoted the nuclear translocation of NRF2. (A)TYMS affected NRF2 expression in nucleus and cytoplasm. (B) RT-qPCR detected the expression of *NRF2* dependent antioxidant enzymes. (C) KEAP1 protein levels in TYMS overexpressed cells, knocked down cells, and correspondence control cells. (D) RT-qPCR detected the expression of *KEAP1* in TYMS overexpressed cells, knocked down cells, and correspondence control cells. (E) TYMS overexpression resulted in a greater accumulation of red fluorescence produced by NRF2 protein in the nucleus. Scale bars: 100 μm. *p < 0.05, **p < 0.01.

The NRF2 pathway is a crucial system for maintaining redox homeostasis in the body. Following a 4-hour treatment with Mg132, the examination of cytoplasmic and nuclear NRF2 protein levels in cells revealed heightened expression in the nucleus of SW480 cells that overexpress TYMS (Figure 4A). Conversely, there was a reduction of NRF2 expression in the cytoplasm. In contrast, HCT116 cells with suppressed TYMS expression showed a reduction in nuclear NRF2 protein levels and an elevation in cytoplasmic NRF2 (Figure 4A). RT-qPCR analysis further demonstrated that NRF2, acting as a transcription factor, promoted the transcription of antioxidant genes such as *GCLC*, *SOD1*, and *SLC7A11* in SW480. (Figure 4B). In SW480 and SW620 cells with TYMS overexpression, the protein levels of KEAP1 were observed to decrease (Figure 4C). The validation of RT-qPCR confirmed that increased TYMS expression resulted in a decrease in *KEAP1* transcription (Figure 4D). Conversely, in HCT116 cells with reduced TYMS expression, both the protein levels and mRNA of *KEAP1* increased (Figure 4C, D). Immunofluorescence assays confirmed that TYMS overexpression resulted in a greater accumulation of red fluorescence produced by NRF2 protein in the nucleus (Figure 4E).

### Inhibiting TYMS enhanced the sensitivity of CRC cells to ferroptosis inducer, erastin

**Figure 5:**
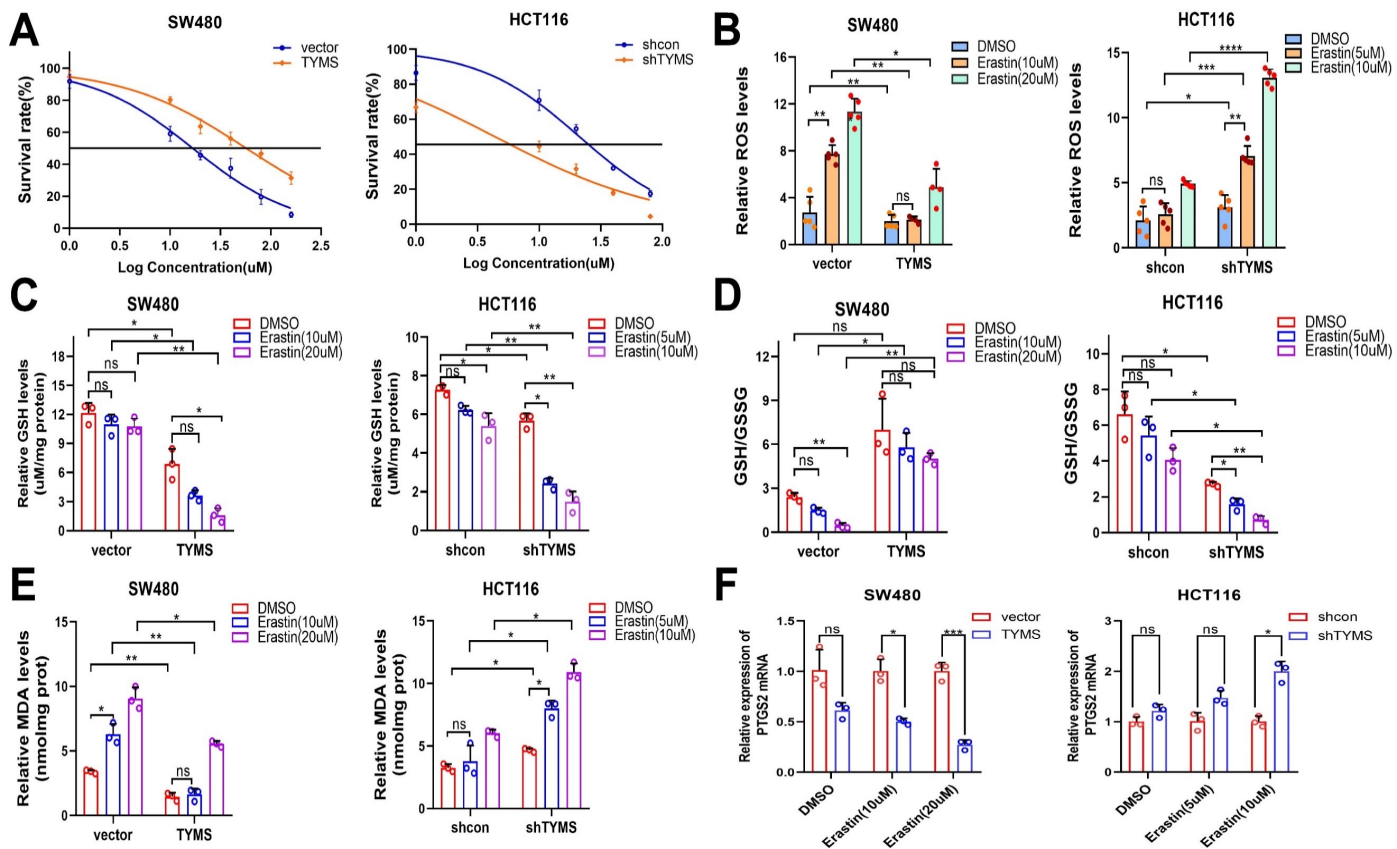
TYMS enhanced resistance to ferroptosis. (A) The IC50s of erastin were analyzed in CRC cells with TYMS overexpress or knockdown. ROS levels (B), GSH levels(C), GSH/GSSG(D), MDA levels(E) and *PTGS2* mRNA levels (F) in TYMS overexpressed cells, knocked down cells, and correspondence control cells treated with different concentrations of erastin. *p < 0.05, **p < 0.01, ***p < 0.001.

NRF2 serves as a pivotal regulatory factor in the mechanism of ferroptosis^20^, ^21^. We postulated that TYMS may impact cell proliferation in CRC cells by enhancing resistance to ferroptosis. This could be achieved through the facilitation of NRF2 nuclear translocation, upregulation of antioxidant gene transcription, augmentation of GSH reserves, reinforcement of cellular antioxidant capacity, and inhibition of intracellular accumulation of ROS. Upon additional treatment of CRC cells with erastin, we observed heightened sensitivity in TYMS-knockdown HCT116 cells compared to shcon, evidenced by lower IC50 values (Figure 5A). Conversely, TYMS overexpression in SW480 cells were more insensitive to erastin (Figure 5A). Colony formation experiments substantiated that the combined downregulation of TYMS and erastin intensified the inhibitory impact on cell growth in HT29 and HCT116 cells, surpassing the effects of individual treatments (Supplementary Figure 2). The combined overexpression of TYMS with erastin exhibited a less noticeable increase in ROS compared to the control group. In contrast, the combined knockdown of TYMS with erastin resulted in a rapid elevation of cellular ROS levels (Figure 5B). Subsequent analysis of the relative GSH levels indicated that the co-treatment of TYMS overexpression with erastin resulted in lesser GSH consumption compared to the control group under the same conditions. Conversely, the combination of TYMS knockdown with erastin led to a rapid depletion of GSH compared to the control group when treated with erastin (Figure 5C).

In SW480 cells overexpressing TYMS, the GSH/GSSG ratio did not exhibit a statistically significant difference compared to the untreated group under the influence of erastin. However, the negative control group showed a significant increase in GSH/GSSG under the same conditions, providing evidence that TYMS overexpression were more resistant to erastin-induced ferroptosis. In contrast, TYMS knockdown in HCT116 cells resulted in a significant decrease in the GSH/GSSG ratio when treated with TYMS in combination with erastin, as compared to the control group under the same conditions with erastin (Figure 5D).

MDA, as one of the end products of lipid peroxidation, serves as an indicator of the level of lipid peroxidation within the organism^22^. The MDA assay revealed that overexpression of TYMS in SW480 cells resulted in lower MDA levels, indicating an enhanced resistance to lipid peroxidation induced by higher concentrations of erastin. Conversely, in HCT116 cells with TYMS knockdown, cells displayed heightened sensitivity to erastin-induced lipid peroxidation (Figure 5E).

In TYMS-overexpressing SW480 cells, the mRNA transcription of *PTGS2* exhibited no statistically significant difference compared to the control group under non-induced erastin conditions. Under erastin induction, SW480 cells overexpressing TYMS demonstrated reduced mRNA transcription levels of *PTGS2* compared to the control group. Likewise, under erastin induction, HCT116 cells with TYMS knockdown exhibited higher mRNA transcription levels of *PTGS2* compared to the control group (Figure 5F).

### The NRF2 inhibitor ML385 reversed the resistance to ferroptosis induced by TYMS

**Figure 6:**
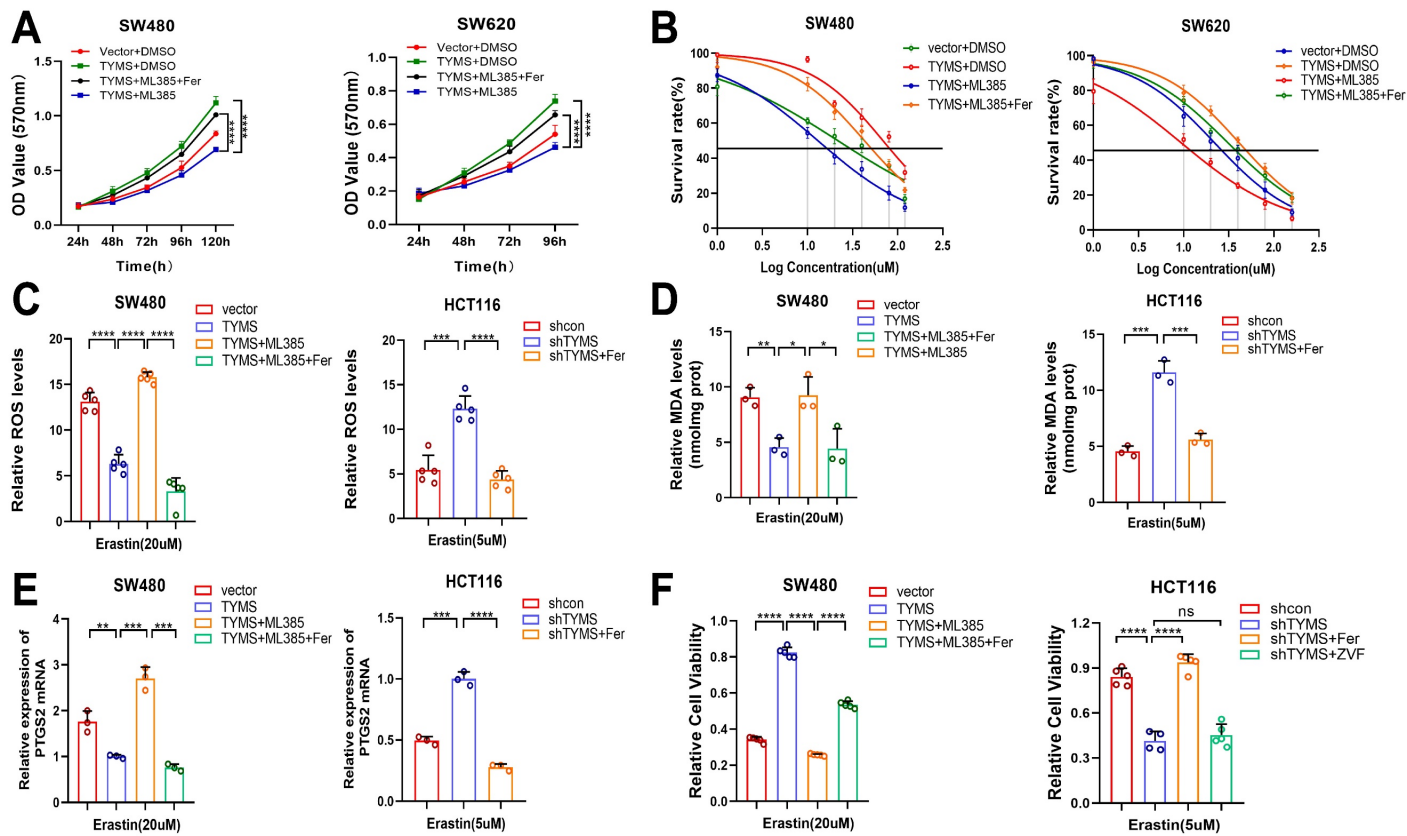
The NRF2 inhibitor ML385 reversed the resistance to ferroptosis. (A) MTT assay was performed after treatment with ML385 and Ferrostatin-1(Fer) in the TYMS-overexpression cells and correspondence control cells. (B) The IC50s of erastin were analyzed after treatment with ML385 and Ferrostatin-1 in the TYMS-overexpression cells and correspondence control cells. ROS levels (C), MDA levels(D), *PTGS2* mRNA levels (E)and cell viability(F) in TYMS overexpressed cells, knocked down cells, and correspondence control cells after treatment with ML385, Ferrostatin-1 and Z-VAD-FMK(ZVF). *p < 0.05, **p < 0.01, ***p < 0.001, ****p< 0.0001.

To investigate the impact of NRF2 on CRC sensitivity to erastin-induced ferroptosis, we designed drug intervention experiments. Utilizing ML385, a novel NRF2 inhibitor^23^, MTT assays illustrated that introducing ML385 counteracted the proliferative effects induced by TYMS overexpression in SW480 and SW620 cells (Figure 6A). Furthermore, the subsequent addition of the ferroptosis inhibitor Ferrostatin-1 restored cell proliferation^24^ (Figure 6A). The IC50 values revealed a notable decrease in resistance to erastin-induced ferroptosis in TYMS-overexpressing SW480 and SW620 cells upon the addition of ML385. Subsequent supplementation with Ferrostatin-1 restored the resistance to ferroptosis (Figure 6B). Similarly, the addition of Ferrostatin-1 in TYMS-knockdown HT29 and HCT116 cells also reinstated resistance to ferroptosis induced by erastin. Upon introduction of ML385, ROS levels increased within TYMS overexpressing SW480 cells, and this effect was counteracted by Ferrostatin-1 (Figure 6C). Ferrostatin-1 successfully mitigated the increased ROS levels observed in HCT116 cells following TYMS knockdown. After supplementation with ML385, there was a significant increase in MDA levels in TYMS-overexpressing SW480 cells. This effect was subsequently reversed upon supplementation with Ferrostatin-1 (Figure 6D). Ferrostatin-1 also reversed the increased MDA levels in HCT116 cells following TYMS knockdown. After the introduction of ML385, TYMS-overexpressing SW480 cells exhibited a notable elevation in mRNA transcription levels of *PTGS2*, which was subsequently reversed upon additional supplementation with Ferrostatin-1 (Figure 6E). Ferrostatin-1 also led to a reduction in *PTGS2* mRNA transcription levels in HCT116 cells with TYMS knockdown. Cell viability assays confirmed enhanced cell viability in TYMS-overexpressing SW480 cells. However, upon the addition of ML385, cell viability markedly decreased, and this effect was subsequently reversed upon supplementation with Ferrostatin-1 (Figure 6F). The reduction in cell viability observed in HCT116 cells following TYMS knockdown was effectively reversed by supplementation with Ferrostatin-1. Notably, the addition of the apoptosis inhibitor Z-VAD-FMK did not reverse the decline in cell viability, supporting the conclusion that TYMS knockdown primarily impacted cell ferroptosis rather than apoptosis (Figure 6F).

### The combined use of 5-FU and erastin enhanced the anti-tumor efficacy in CRC

**Figure 7:**
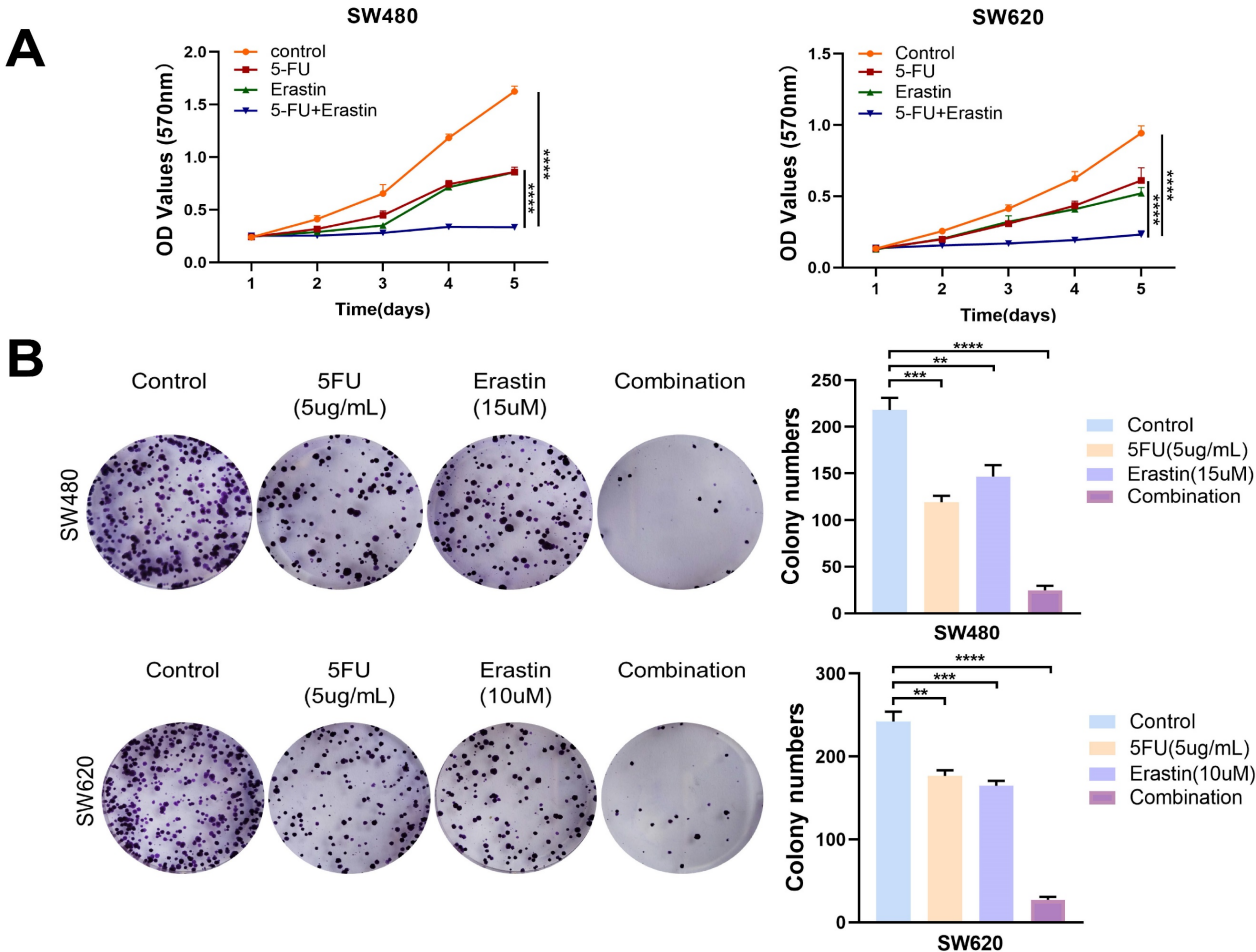
Combination of erastin with 5-FU enhanced anti-tumor effects. (A) MTT assays showed combination of erastin with 5-FU treatment exhibited higher inhibitory effects on cell growth compared with mono treatment in SW480 and SW620. (B) Combination of erastin with 5-FU treatment exhibited higher inhibitory effects on clone formation compared with mono treatment in SW480 and SW620. **p < 0.01, ***p < 0.001, ****p< 0.0001.

5-FU plays a crucial role as an inhibitor of TYMS. In this study, we aimed to explore the potential synergistic antitumor effects of combining 5-FU with erastin. Through MTT assays and colony formation experiments, the results indicated that the combined use of 5-FU and erastin efficiently suppressed the proliferation of CRC cells, demonstrating a synergistic anti-tumor effect (Figure 7A, B). This offered additional choices for multi-targeted chemotherapy based on 5-FU.

## Discussion

CRC commonly presents with subtle clinical manifestations and is frequently diagnosed at advanced stages. With the increasing occurrence of CRC in younger individuals, there is a tendency for these patients to underestimate the importance of early screening^25^. This oversight greatly hinders the efficacy of CRC treatment. At present, early diagnosis of CRC relies primarily on a preliminary screening involving fecal occult blood test and two tumor markers, CEA and CA19-9^26^. The final confirmation is made through colonoscopy. However, the sensitivity of these two tumor markers in detecting stage I and II patients is below 40%. Establishing a multi-target combined detection model is both necessary and urgent to overcome this limitation.

Through immunohistochemistry, it has been found that the immunohistochemical score of TYMS in colorectal tumor tissue was notably higher than that in adjacent tissue. Furthermore, it was worth noting that, possibly due to constraints in sample size, the high expression of TYMS did not necessarily result in a poorer prognosis compared to patients with low expression. Unexpectedly, among patients who did not receive chemotherapy, those with high TYMS expression exhibited a poorer prognosis compared to those with low expression. This suggested that TYMS could be considered as a prognostic marker for patients who have not undergone chemotherapy. In the clinical-pathological correlation analysis, a correlation was found between TYMS and tumor size as well as tumor N staging. This indicated that TYMS was a crucial factor influencing the malignant progression of tumors.

The increased expression of TYMS in CRC tissue implied that TYMS plays a role in influencing the biological behavior of the tumor. Earlier investigations had validated the oncogenic function of TYMS in CRC. To explore deeper insights into the molecular mechanisms driving TYMS-induced carcinogenesis, we utilized metabolomics to identify differential metabolites in SW480 cells overexpressing TYMS. The results indicated an increased level of GSH in SW480 cells with TYMS overexpression, a finding subsequently confirmed in cell experiments. NRF2 regulates the synthesis of GSH^27^. Initially, NRF2 oversees the important protein GCLC and the xCT subunit of the xc- system, both integral in the rate-limiting step of GSH synthesis. Moreover, NRF2 also controls the expression of glutathione peroxidase (GPX) 2 and glutathione reductase (GSR) 1. GPX 2 generates GSSG during the peroxide reduction process, while GSR1 reduces GSSG, thereby ensuring the maintenance of intracellular GSH levels. Endogenously produced ROS, such as hydrogen peroxide, undergo reduction by GSH through the action of GPX. In this process, GSH is oxidized to produce GSSG. We detected NRF2, which is responsible for regulating ROS levels and GSH synthesis. Following TYMS overexpression, we observed an increased accumulation of NRF2 in the cell nucleus, accompanied by downregulation of KEAP1, the ligand for NRF2. In the body, the double glycine repeat (DGR) region of the KEAP1 protein typically binds to NRF2, forming a complex that links to the E3 complex. This facilitates the transfer of ubiquitin from E3 to lysine residues on NRF2, leading to the rapid degradation of ubiquitinated NRF2. KEAP1 contains several highly reactive cysteines, making it an efficient and sensitive redox sensor^28^. Following covalent modification by electrophilic molecules, its targeting to the NRF2 protein for proteasomal degradation is blocked, resulting in the nuclear accumulation of NRF2 protein. Following the degradation, mutation, or downregulation of the KEAP1 protein, NRF2 may translocate into the nucleus, conferring a survival advantage to tumor cells. KEAP1 is the third most frequently mutated gene in LUAD^29^, ^30^. Rodrigo demonstrated this by constructing a Kras-driven KEAP1-mutant LUAD mouse model, revealing that KEAP1 loss results in the overactivation of NRF2^31^. A sulfonamide-containing drug can disrupt the protein-protein interaction between KEAP1 and NRF2, promoting NRF2 nuclear translocation, restoring GSH levels, and subsequently alleviating lung inflammation^32^. Enhancing the interaction between KEAP1 and NRF2 at the protein level holds promise as a valuable approach in cancer therapy. In our research, the overexpression of TYMS in CRC cells led to a reduction in KEAP1 expression, promoting the translocation of NRF2 into the nucleus. Subsequently, this led to an upregulation of downstream antioxidant genes, reprogramming of CRC cell metabolism, elevation in intracellular GSH levels, reduction in intracellular ROS levels, and decrease in the generation of 4-hydroxyhexenal, a product from lipid peroxidation.

Ferroptosis represents a distinctive form of cell death, marked by alterations in factors including ROS, levels of lipid peroxidation, the balance of glutathione (GSH/GSSG), and the expression of *PTGS2*. NRF2 stands out as a crucial factor in regulating lipid peroxidation and ferroptosis. Numerous studies have confirmed that the expression of NRF2 imparts resistance to ferroptosis. In the research conducted by Jin Feng^33^, it was revealed that Ibrutinib could heighten the sensitivity of CRC cells to ferroptosis inducers by inhibiting the nuclear translocation of NRF2. In Bei's study^34^, the E-prostanoid 1 receptor (EP1) was identified as a protective element for myocardial cells against doxorubicin-induced ferroptosis. This protection occurred through the activation of antioxidant gene expression driven by NRF2. We hypothesized that the overexpression of TYMS may enhance the antioxidant capacity of colorectal cancer (CRC) cells by facilitating the nuclear translocation of NRF2, thereby strengthening the resistance of CRC cells to ferroptosis. In initial drug sensitivity experiments with erastin, it was observed that overexpressing TYMS demonstrated greater resistance to cell death induced by erastin compared to the control group. Conversely, cells with reduced TYMS expression exhibited heightened sensitivity to erastin, likely attributed to the decrease in GSH levels following TYMS knockdown. We conducted additional assessments of GSH levels in various concentrations of erastin-treated groups. Our findings indicated that GSH depletion occurred at lower concentrations of erastin in TYMS knockdown CRC cells compared to control cells. In contrast, CRC cells overexpressing TYMS were able to sustain elevated levels of GSH even when subjected to higher concentrations of erastin treatment compared to the control group. Through a comparative analysis of key markers of ferroptosis, including GSH/GSSG, *PTGS2* mRNA expression, and MDA levels, it was evident that overexpress TYMS displayed enhanced resistance to ferroptosis induced by relatively high concentrations of erastin. The resistance to ferroptosis conferred by TYMS overexpression was reversed upon blockade with the NRF2 inhibitor ML385. Subsequent supplementation with the ferroptosis inhibitor Ferrostatin-1 restored the resistance.

5-FU is a classical inhibitor of TYMS, which was first synthesized by Duschinsky and colleagues in 1957^35^. In the same year, Curreri Ausfield pioneered its clinical application^36^. The primary anticancer mechanism of 5-FU involves the metabolic transformation of 5-FU into 5-fluoro-deoxyuridine monophosphate (5-FdUMP) within the body. 5-FdUMP competes with dUMP for binding sites on TYMS, creating a stable ternary complex with the coenzyme factor 5,10-CH2-THF. This complex inhibits TYMS's catalytic activity in converting dUMP to dTMP, leading to the interruption of DNA replication and cell death. Currently, 5-FU is extensively used in chemotherapy for various cancers, including CRC. However, the efficacy of 5-FU chemotherapy is not entirely satisfactory due to tumor heterogeneity, which enables the survival and extensive proliferation of drug-resistant tumor cells during treatment. Research findings demonstrated that in 5-FU-resistant cells, there was an upregulation in *TYMS* mRNA levels, resulting in increased catalytic activity of thymidylate synthase^37^. Furthermore, studies indicated the involvement of specific microRNAs in regulating resistance following 5-FU chemotherapy^38^. The synergistic effect of combining drugs targeting multiple pathways has been shown to be an effective strategy in overcoming chemotherapy resistance. Experimental evidence from MTT assays and clonogenic formation studies validated the synergism achieved by combining erastin with the TYMS inhibitor 5-FU. This discovery offers valuable insights for developing multi-targeted combination therapies based on the foundation of 5-FU.

## Conclusion

In summary, our findings indicated that TYMS could enhance the antioxidant capability of CRC cells through the KEAP1-NRF2 pathway, thereby increasing their resistance to ferroptosis. TYMS knockdown sensitized CRC cells to erastin-induced ferroptosis. Furthermore, the observed synergistic anti-tumor effect of combining the ferroptosis inducer erastin with the TYMS inhibitor 5-FU provided valuable insights for multi-targeted chemotherapy.

## Supplementary Material

Supplementary figures and table.

## Figures and Tables

**Table 1:** Associations between TYMS expression and clinicopathological parameters of CRC patients.

Parameters	Number	High expression	Low expression	χ^2^	*P*
Overall	150	59	91		
Gender					
Male	86 (57.3)	33 (55.9)	53 (58.2)	0.078	0.780
Female	64 (42.7)	26 (44.1)	38 (41.8)		
Age (years)					
≤60	68 (45.3)	24 (40.7)	38 (41.8)	0.017	0.896
>60	82 (54.7)	35 (59.3)	53 (58.2)		
T classification					
T1-2	34 (22.7)	9 (15.3)	25 (27.5)	3.048	0.081
T3-4	116 (77.3)	50 (84.7)	66 (72.5)		
N classification					
N0	82 (54.7)	25 (42.4)	57 (62.6)	5.931	0.015*
N1-2	68 (45.3)	34 (57.7)	34 (37.4)		
AJCC stage					
I and II	80 (53.3)	24 (40.7)	56 (61.5)	6.258	0.012*
III and IV	70 (46.7)	35 (59.3)	35 (38.5)		
Status					
Living	80 (53.3)	27 (45.8)	53 (58.2)	2.709	0.100
Death	52 (34.7)	25 (42.4)	27 (29.7)		
Unknown	18 (12.0)	7 (11.9)	11 (12.1)		
CEA					
Negative	99 (66.0)	41 (69.5)	58 (63.7)	0.528	0.467
Positive	51 (34.0)	18 (30.5)	33 (36.3)		
CA19-9					
Negative	136 (90.7)	56 (94.9)	80 (87.9)	1.329	0.249
Positive	14 (9.3)	3 (5.1)	11 (12.1)		
Tumor size					
≤5cm	88 (58.7)	27 (45.8)	61 (67.0)	6.678	0.010*
>5cm	62 (41.3)	32 (54.2)	30 (33.0)		
BRAF^V600E^					
Negative	114 (76.0)	49 (83.1)	65 (71.4)	2.651	0.104
Positive	36 (24.0)	10 (16.9)	26 (28.6)		
HER-2					
Negative	109 (72.7)	41 (69.5)	68 (74.7)	0.494	0.482
Positive	41 (27,3)	18 (30.5)	23 (25.3)		
MSI					
Negative	135 (90.0)	53 (89.8)	82 (90.1)	0.003	0.956
Positive	15 (10.0)	6 (10.2)	9 (9.9)		
Nerve invasion					
Negative	135 (90.0)	52 (88.1)	83 (91.2)	0.376	0.540
Positive	15 (10.0)	7 (11.9)	8 (8.8)		
Vascular invasion					
Negative	132 (88.0)	51 (86.4)	81 (89.0)	0.224	0.636
Positive	18 (12.0)	8 (13.6)	10 (11.0)		
Distal metastasis					
Negative	147 (98.0)	57 (96.6)	90 (98.9)	-	0.562
Positive	3 (2.0)	2 (3.4)	1 (1.1)		
Smoking					
Yes	47 (31.3)	17 (28.8)	30 (33.0)	0.287	0.592
NO	103 (68.7)	42 (71.2)	61 (67.0)		
Drinking					
Yes	28 (18.7)	9 (15.3)	19 (20.9)	0.746	0.388
No	122 (81.3)	50 (84.7)	72 (79.1)		

Note: All values are number (%); *p < 0.05
