# Supplementary material for: TYMS Enhances Colorectal Cell Antioxidant Capacity Via the KEAP1-NRF2 Pathway to Resist Ferroptosis
Source: J Cancer. 2025 Jan 1;16(2):417–29. doi: 10.7150/jca.102931 (PMC11685691; doi:10.7150/jca.102931)
Supplement: Supplementary file 1 — Supplementary figures and table. [file jcav16p0417s1.pdf]

Table S1 Primers used in the study.

No.	Target gene		Sequence
1	*GAPDH*	Forward Primer	GGAGCGAGATCCCTCCAAAAT
		Reverse Primer	GGCTGTTGTCATACTTCTCATGG
2	*TYMS*	Forward Primer	AACCCTGACGACAGAAGA
		Reverse Primer	CGATGTTGAAAGGCACA
3	*NRF2*	Forward Primer	GCGACGGAAAGAGTATGAGC
		Reverse Primer	GGGAGTAGTTGGCAGATCCA
4	*KEAP1*	Forward Primer	TGCGCTGCGAGTCCGAGGTCTTC
		Reverse Primer	TCGAAGATCTTGACCAGGTAGT
5	*GCLC*	Forward Primer	ACGGAGGAACAATGTCCGAG
		Reverse Primer	TGTGAACCCAGGACAGCCTA
6	*SOD1*	Forward Primer	GAAGGTGTGGGGAAGCATTA
		Reverse Primer	ACATTGCCCAAGTCTCCAAC
7	*SOD2*	Forward Primer	GCTGGAAGCCATCAAACG
		Reverse Primer	TTAGAACAAGCGGCAATCTG

(A)

(B)

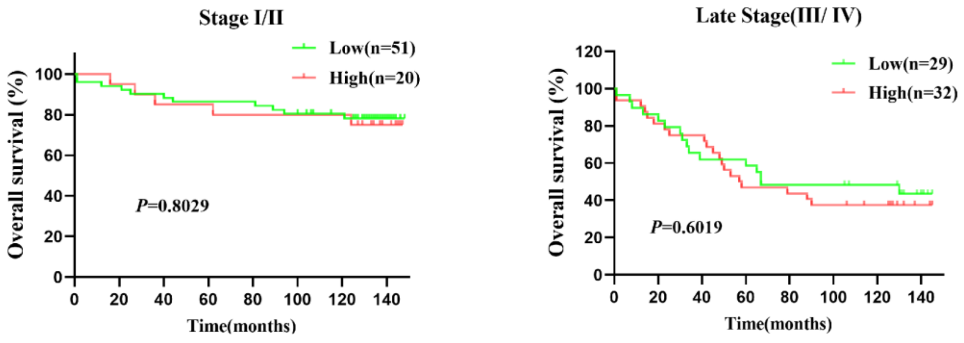

Figure S1. The expression levels of TYMS did not impact overall survival in patients with stage I/II(A) or stage III/IV (B).

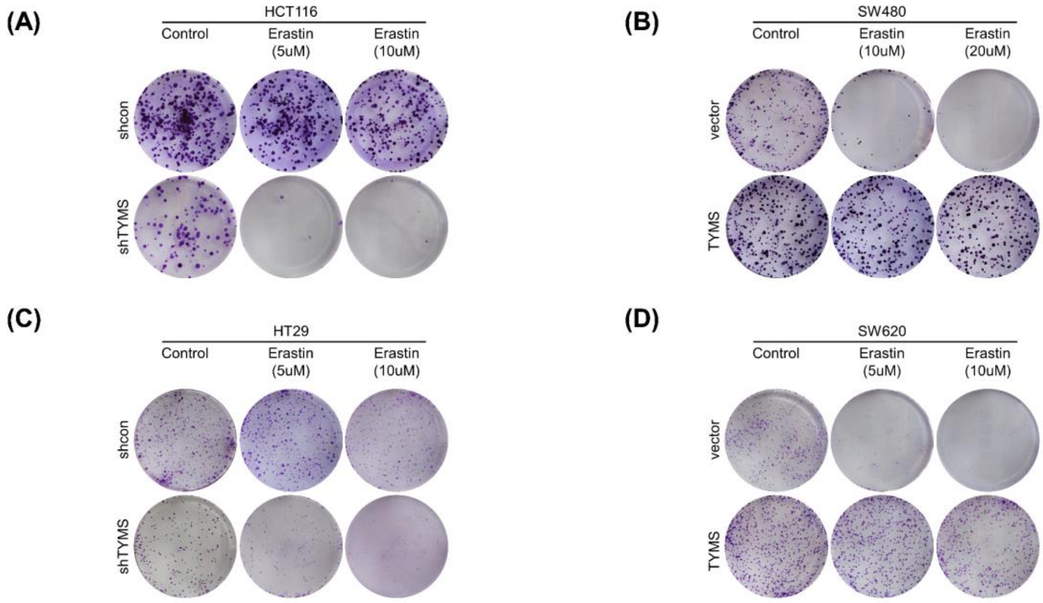

Figure S2. Effect of TYMS expression levels on clony formation under different concentrations of erastin.
